# Efficient near-ultraviolet (NUV) electroluminescence based on a benzonitrile acceptor HLCT material with balanced carrier mobilities and high color purity†

**DOI:** 10.1039/d5sc03458b

**Published:** 2025-07-17

**Authors:** Li Zhang, Chenglin Ma, Xin Wang, Yannan Zhou, Jingru Song, Mizhen Sun, Qikun Sun, Shi-Tong Zhang, Wenjun Yang, Shanfeng Xue

**Affiliations:** a State Key Laboratory of Advanced Optical Polymer and Manufacturing Technology, Qingdao University of Science and Technology 53-Zhengzhou Road Qingdao 266042 PR China sfxue@qust.edu.cn; b Shandong Engineering Research Center of Green and High-value Marine Fine Chemical, Shandong Peninsula Blue Economy and Engineering Research Institute, Weifang University of Science and Technology Shouguang 262700 PR China; c State Key Laboratory of Supramolecular Structure and Materials, Department of Chemical Engineering and Applied Chemistry, College of Chemistry, Jilin University Changchun 130012 PR China

## Abstract

Balanced carrier injection/transport and high color purity are critical for highly efficient near-ultraviolet (NUV) electroluminescence (EL) emitters. Herein, two novel structurally simple NUV donor–π–acceptor (D–π–A) molecules, namely CZ-PPCN and TPA-PPCN, with a benzene π-bridge containing carbazole/triphenylamine as donors and benzonitrile as the acceptor were designed and synthesized, and they exhibited hybrid local charge transfer (HLCT) characteristics. The introduction of the benzonitrile group of the CZ-PPCN molecule not only enhances charge carrier injection but also promotes hole and electron mobility balance through the formation of supramolecular hydrogen bonds (both up to 10^−5^ cm^2^ V^−1^ s^−1^). The non-doped and doped devices fabricated using CZ-PPCN exhibit the best excellent EL performances. As a result, the non-doped device of the CZ-PPCN emitter exhibits a high maximum external quantum efficiency (EQE_max_) of 6.42%, and the device exhibits deep-blue emission with Commission International de L'Eclairage (CIE) coordinates of (0.154, 0.075). More importantly, the CZ-PPCN-based doped device exhibits EL emission peaks at 405 nm with CIE coordinates of (0.159, 0.040), while still retaining a high EQE of 7.14%. This work provides a simple design strategy for advancing next-generation NUV emitters with balanced carrier mobility and superior color purity.

## Abstract 2

Simple-structured NUV molecule with a benzonitrile acceptor achieves rapid and balanced carrier mobility. The doped OLED shows high efficiency and superior color purity, leading similar devices based on the HLCT mechanism (400 nm ≤ *λ*_EL_ ≤ 410 nm).

## Graphical abstract

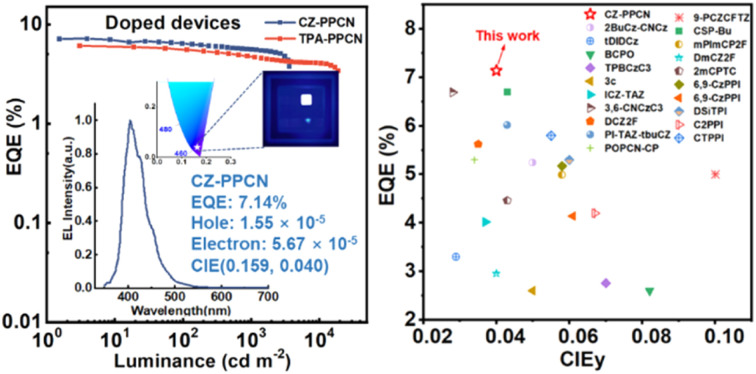

## Introduction

Organic light-emitting diodes (OLEDs), as a next-generation display and lighting technology, have garnered significant attention in both academia and industry.^1–4^ Nowadays, high efficiency near-ultraviolet (NUV) OLEDs are expected to become a critical milestone in advancing OLED display technology, not only owing to their specific application in lithography, sterilization and high-density information storage but also on account of their capability as energy donors to achieve full spectral range coverage of visible light and their significant reduction of energy consumption in lighting systems.^5–9^ But compared to red, green, and blue (RGB) OLEDs, NUV emitters exhibit significantly wider bandgaps due to their requirement for higher energy.^10–12^ The inherently wide highest occupied molecular orbital (HOMO)/lowest unoccupied molecular orbital (LUMO) bandgaps of these materials always induce a significant imbalance in carrier injection and transport efficiency, and thereby will make it difficult for the device efficiency to reach a high level.^13–15^ Meanwhile, the color purity of NUV light sources still critically restricts their development. The reports on efficient blue-NUV materials meeting the CIE coordinates (0.131, 0.046) specified by the International Telecommunication Union Radiocommunication (ITU-R) Sector Recommendation BT.2020 for Ultra-High-Definition Television (UHDTV) blue standards still remain scarce.^16–20^

In 2012, Ma *et al.* first proposed the “hybridized local and charge-transfer excited state (HLCT)”, which synergistically integrates the advantages of both the locally excited (LE) state and charge-transfer (CT) state.^21^ Specifically, the low-lying LE-emissive state guarantees short-wavelength emission, thus ensuring color purity in devices, while the CT component promotes the reverse intersystem crossing process from higher-lying triplet states (hRISC), thereby achieving mitigated efficiency roll-off at a high-brightness level.^22–25^ Emitters based on the HLCT mechanism demonstrate promising potential for simultaneously attaining high photoluminescence quantum yields (PLQYs), short-wavelength emission, and enhanced exciton utilization efficiency. The construction of donor–acceptor (D–A) or D–π–A molecular architecture has been demonstrated as an effective strategy to enhance charge injection and transport capabilities.^26–31^ Ma *et al.* reported a NUV molecular mTPA-PPI with a D–A structure, realizing a maximum EQE of 3.33%, with CIE coordinates of (0.161, 0.049).^32^ In 2021, Lu *et al.* reported a novel NUV emitter with a D–A construction, namely CSiTPI, and the corresponding non-doped device exhibits a remarkable EQE of 7.1% with CIE_*y*_ of 0.06.^33^ In 2022, Cui *et al.* developed a violet-blue fluorescent material featuring a D–π–A structure, and the resulting device achieves high color purity with CIE_*y*_ of 0.046; the EQE is only 4.1%.^34^ Therefore, developing novel NUV molecules with high EQE and high color purity remains a highly promising and challenging task.

In this work, we propose a straightforward molecular design strategy based on the HLCT mechanism, and utilizing benzonitrile as the acceptor and high hole-mobility carbazole/triphenylamine as donors, two novel emitters 4′-(9-phenyl-9*H*-carbazol-3-yl)-[1,1′-biphenyl]-4-carbonitrile (CZ-PPCN) and 4′′-(diphenylamino)-[1,1′:4′,1′′-terphenyl]-4-carbonitrile (TPA-PPCN) have been designed and synthesized. The introduction of a cyano group can effectively lower the LUMO energy level in OLEDs, thereby reducing the electron injection barrier.^35,36^ Carbazole and triphenylamine donors introduce spatial barriers to suppress tight π–π stacking, effectively mitigating exciton quenching. And the benzene bridge increases the donor–acceptor distance, preventing excessive CT excited-state formation. Theoretical calculations and photophysical studies confirm the HLCT characteristics of these emitters. Non-doped devices based on CZ-PPCN and TPA-PPCN achieved EQEs of 6.42% and 4.16%, with maximum luminances of 12 835 cd m^−2^ and 27 090 cd m^−2^, respectively. Notably, CZ-PPCN exhibited balanced carrier mobilities (*μ*_h_ = 1.55 × 10^−5^ cm^2^ V^−1^ s^−1^; *μ*_e_ = 5.57 × 10^−5^ cm^2^ V^−1^ s^−1^). Further optimization through doping these emitters into a CzSi host matrix significantly improved device performance. The doped devices achieved enhanced EQEs of 7.14% (CZ-PPCN) and 6.07% (TPA-PPCN) with exceptional color purity reflected in CIE coordinates of (0.159, 0.040) and (0.176, 0.105), respectively. Extraordinarily, CZ-PPCN achieved an exceptionally low CIE_*y*_ coordinate of 0.04 while maintaining high device efficiency. These results highlight the efficacy of the HLCT-based molecular design in balancing carrier transport and high color purity for high-performance NUV-OLEDs.

## Results & discussion

### Synthesis and characterization

**Scheme 1:**
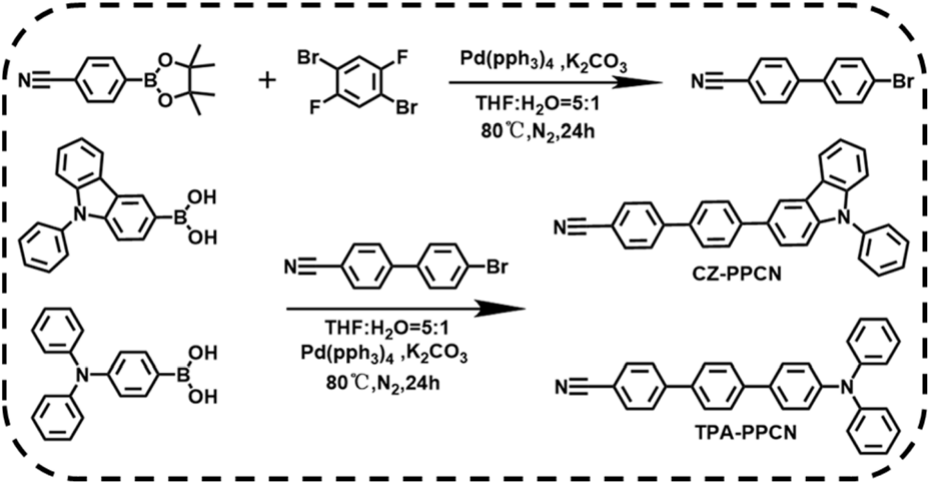
Synthetic routes and chemical structures of CZ-PPCN and TPA-PPCN.

The synthetic routes of CZ-PPCN and TPA-PPCN are shown in Scheme 1 and the detailed synthesis steps are shown in the “Synthesis and routines” section of the ESI (Fig. S1†). The target molecules were prepared by Suzuki cross-coupling reactions with high yields of 80% and 79%, respectively. And they were both fully characterized by ^1^H NMR, ^13^C NMR and high-resolution mass spectrometry (Fig. S2–S4†).

### Theoretical calculations

**Fig. 1:**
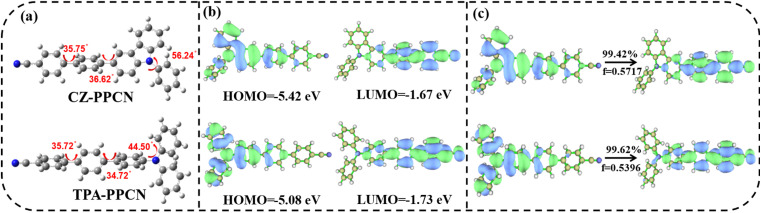
(a) Optimized molecular geometry; (b) the frontier molecular orbital distribution; (c) the S_0_ →S_1_ natural transition orbitals (NTOs) of CZ-PPCN and TPA-PPCN. The *f* is the oscillator strength.

To gain deeper insights into the ground- and excited-state properties of these molecules, we performed density functional theory (DFT) and time-dependent DFT (TDDFT) calculations to analyze their spatial electron cloud distributions. As can be seen from the optimized geometric configurations, the two molecules exhibit similar torsional degrees, particularly between the acceptor and biphenyl bridge (Fig. 1a), and thus the HOMO/LUMO of the two materials display similar separated orbital patterns. For CZ-PPCN, the LUMO is predominantly localized on the benzonitrile acceptor and π-bridge, with partial delocalization onto the donor-linked benzene ring, while the HOMO is primarily distributed over the carbazole donor and π-bridge, with minimal contribution from the acceptor's benzene ring. In contrast, TPA-PPCN exhibits similar LUMO/HOMO distributions but differs in that its HOMO shows no delocalization onto the acceptor moiety. These observations reveal that both molecules exhibit HLCT characteristics, as evidenced by the partial overlap and spatial separation of their HOMOs and LUMOs (Fig. 1b). Further analysis of the natural transition orbital (NTO) for the first excited state (S_1_) demonstrates that the NTO distributions of both emitters closely resemble their respective frontier orbital patterns, reinforcing the HLCT nature. As illustrated in Fig. 1c, the hole and electron components of the NTOs in CZ-PPCN and TPA-PPCN are spatially separated yet partially overlapping, consistent with the HLCT mechanism that synergistically combines localized exciton radiation and charge-transfer-mediated reverse intersystem crossing (RISC).

### Single crystal analysis

**Fig. 2:**
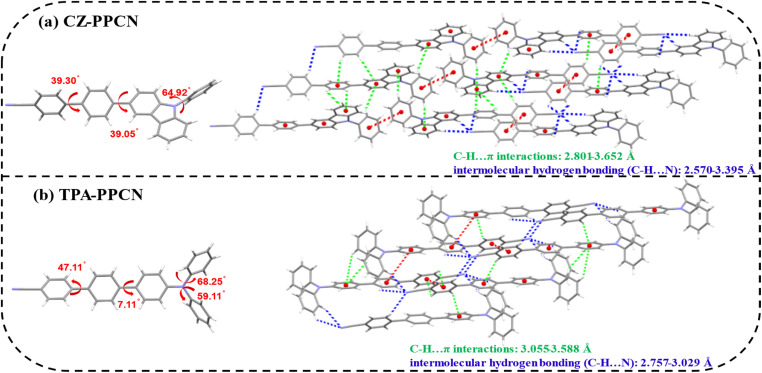
Crystal structures and packing patterns of (a) CZ-PPCN and (b) TPA-PPCN.

To elucidate the molecular packing patterns and intermolecular interactions, single crystals of both compounds were grown *via* a temperature-gradient sublimation method. Crystallographic analysis (as shown in Fig. 2) reveals dihedral angles between the acceptor and benzene bridge of 39.3° (CZ-PPCN) and 49.11° (TPA-PPCN), with torsional angles between the donor (carbazole/triphenylamine) and benzene bridge measuring 39.05° and 7.11°, respectively. The twisted conjugation bridges reduce the effective π-conjugation length in the D–π–A framework, enabling precise color purity, while the bulky donors introduce steric-hindrance to prevent tight π–π stacking, thereby suppressing exciton quenching. Notably, the cyano group facilitates multiple C–H⋯N hydrogen bonds, which restrict intramolecular rotation and enhance photoluminescence efficiency. The combination of moderate π–π interactions and abundant hydrogen bonds promotes ordered molecular packing, creating continuous pathways for rapid carrier transport during electroluminescence.^37,38^ CZ-PPCN and TPA-PPCN share similar linear architectures and packing pattens. But compared to TPA-PPCN, the donor moiety of CZ-PPCN participates in π–π stacking interactions, which facilitate efficient hole transport. However, the twisted spatial configuration of triphenylamine in TPA-PPCN results in larger intermolecular distances, leading to weaker intermolecular interactions. Conversely, CZ-PPCN exhibits abundant C–H⋯π interactions (2.801–3.652 Å), enabling the formation of multiple hole and electron transport channels and achieving balanced carrier mobility. For TPA-PPCN, the hydrogen bonding network is spatially confined (range: 2.757–3.029 Å), with interactions being more localized and directional. This concentrated bonding pattern likely promotes the formation of well-ordered electron transport pathways, effectively reducing energy barriers for charge hopping and contributing to its higher electron mobility. These distinct intermolecular interaction landscapes directly correlate with their divergent charge transport behaviors in OLED devices.

### Photophysical properties

**Fig. 3:**
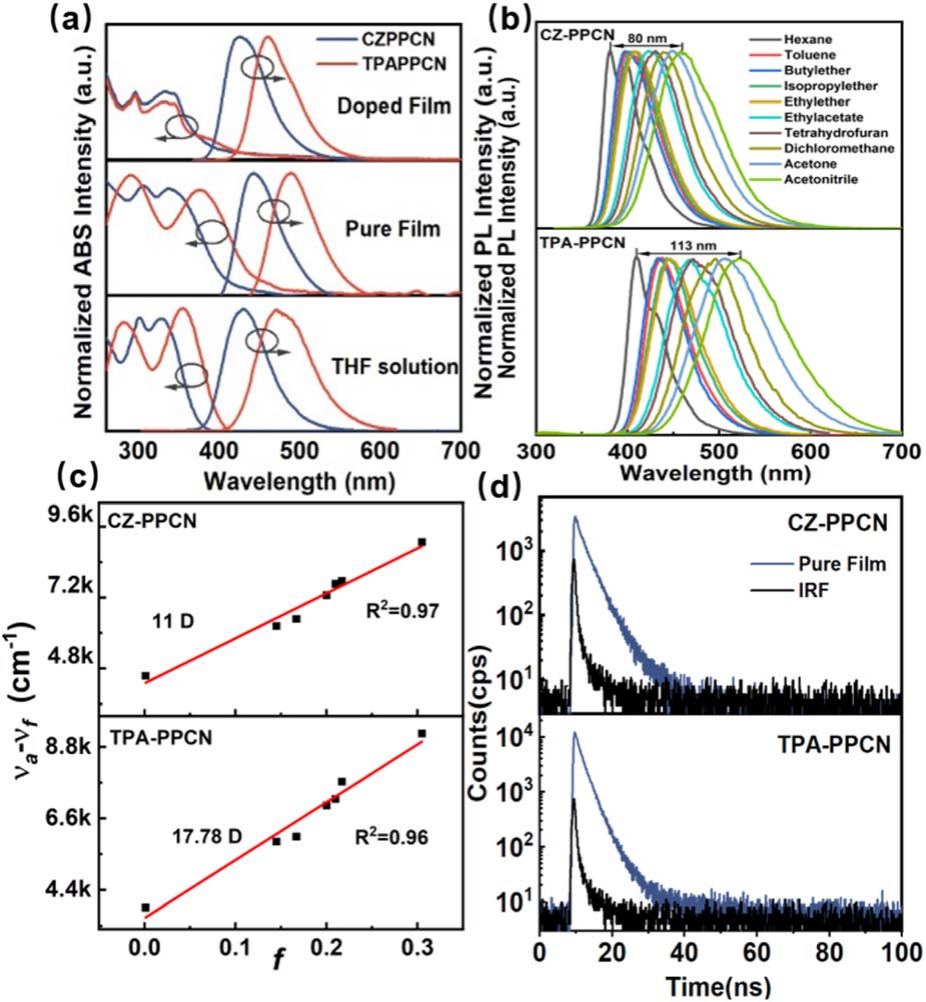
(a) The UV-vis absorption spectra and the PL spectra of CZ-PPCN and TPA-PPCN in THF solution, neat films and the doped film, respectively. (b) Solvation spectra in different polar solvents. (c) Lippert–Mataga model depicting the correlation between Stokes shift and solvent polarity. (d) Transient spectrum of neat films.

The ultraviolet-visible (UV-vis) absorption spectra and photoluminescence (PL) spectra of both molecules in various states are presented in Fig. S8.† For CZ-PPCN, the absorption bands remain consistent across different states: the peak at 300 nm corresponds to the π → π* transition of the carbazole moiety, while the broad absorption between 330 and 340 nm arises from the parallel and transverse transitions of the carbazole-phenyl group. In contrast, TPA-PPCN exhibits a characteristic backbone absorption below 300 nm, with the 330–380 nm region dominated by π–π* transitions of the triphenylamine donor. Notably, both molecules show slight emission red-shifts in thin films compared to in tetrahydrofuran (THF, 10^−5^ M). However, when doped into a host matrix, their emission spectra blue-shift, likely due to restricted molecular rotation in the solid state, which reduces excited-state relaxation (Fig. 3a).

Further investigation of solvent-dependent absorption and emission properties reveals that the emission wavelengths of both emitters red-shift with increasing solvent polarity (Fig. 3b), indicating the presence of the CT component in S_1_. TPA-PPCN, with its stronger electron-donating triphenylamine group and extended conjugation, exhibits a higher CT-state contribution, resulting in less desirable color purity and more pronounced solvatochromic shifts. To quantify these effects, the Lippert–Mataga solvent model was employed to correlate Stokes shifts with solvent polarity (Fig. 3c). Both CZ-PPCN and TPA-PPCN display linear relationships across low-to high-polarity solvents, with dipole moments of 11 D and 17.78 D, respectively. The single-slope linearity suggests a quasi-degenerate hybridization of LE and CT states in the excited state, consistent with the HLCT mechanism.^39,40^ Furthermore, we analyzed the low-temperature (LT) fluorescence (FL) and phosphorescence (Ph) of both molecules in tetrahydrofuran (THF, 10^−5^ M) solution at 77 K (Fig. S9†). Based on the 0–0 emission peaks in the LT spectra, the lowest singlet and triplet energy levels were determined to be 3.02/2.56 eV (CZ-PPCN) and 2.66/2.29 eV (TPA-PPCN), corresponding to singlet–triplet energy gaps (Δ*E*_ST_) of 0.46 eV and 0.37 eV, respectively. These large Δ*E*_ST_ values indicate that RISC between T_1_ and S_1_ is thermodynamically prohibited. Transient emission measurements across solvents of varying polarities revealed only nanosecond-timescale components for both emitters (Fig. S10†). In neat films, single-exponential decay profiles were observed, with lifetimes (*τ*) of 3.413 ns (CZ-PPCN) and 5.549 ns (TPA-PPCN), consistent with the absence of delayed fluorescence (Fig. 3d). These results collectively confirm the HLCT state characteristics of the emitters, where the coexistence of localized and charge-transfer excited states enables efficient radiative recombination without triplet state involvement. Remarkably, both CZ-PPCN and TPA-PPCN exhibit a high PLQY of 91.5% (THF solution), 80% (neat film) and 97.8% (THF solution), and 80% (neat film), respectively (Table S3†). This exceptional efficiency is attributed to their rigid molecular frameworks and extensive intermolecular interactions, which suppress structural vibrational relaxation and minimize the probability of non-radiative transitions during excitation.

### Thermal and electrochemical properties

Excellent thermal properties are indispensable for the OLED manufacturing process, particularly vacuum deposition technology. To investigate the thermal stabilities of molecules, the thermal properties of CZ-PPCN and TPA-PPCN were tested through thermogravimetric analysis (TGA) and differential scanning calorimetry (DSC) under a nitrogen atmosphere, and the relevant data are presented in the ESI.† As shown in Fig. S11,† the thermal decomposition temperatures (*T*_d_, corresponding to 5% weight loss) of CZ-PPCN and TPA-PPCN were 369 °C and 344 °C, respectively which implies that molecules have excellent thermal stabilities. In accordance with the DSC curves (Fig. S12†), there was no glass transition temperature (*T*_g_) of CZ-PPCN indicating its better morphological stability under heating.

The electrochemical properties of both molecules were investigated *via* cyclic voltammetry (CV) to guide the selection of functional layers in device structure optimization (Fig. S13†). CV measurements against the ferrocene/ferrocenium (Fc^+^/Fc) redox couple revealed oxidation onsets of 0.93 V (CZ-PPCN) and 0.69 V (TPA-PPCN), with reduction onsets at −2.11 V and −2.18 V, respectively. The HOMO and LUMO energy levels were derived using the empirical equation (Formula (S1)†). Consequently, CZ-PPCN exhibited a HOMO of −5.53 eV and LUMO of −2.61 eV, while TPA-PPCN showed a HOMO of −5.28 eV and LUMO of −2.54 eV. The calculated electrochemical bandgaps (*E*_g_) were 2.92 eV (CZ-PPCN) and 2.74 eV (TPA-PPCN), indicating a narrower bandgap for TPA-PPCN that aligns with its red-shifted emission compared to CZ-PPCN. These results provide critical guidance for tailoring charge-transport layers to match energy-level alignments in OLED architectures.

### Carrier transport properties

**Fig. 4:**
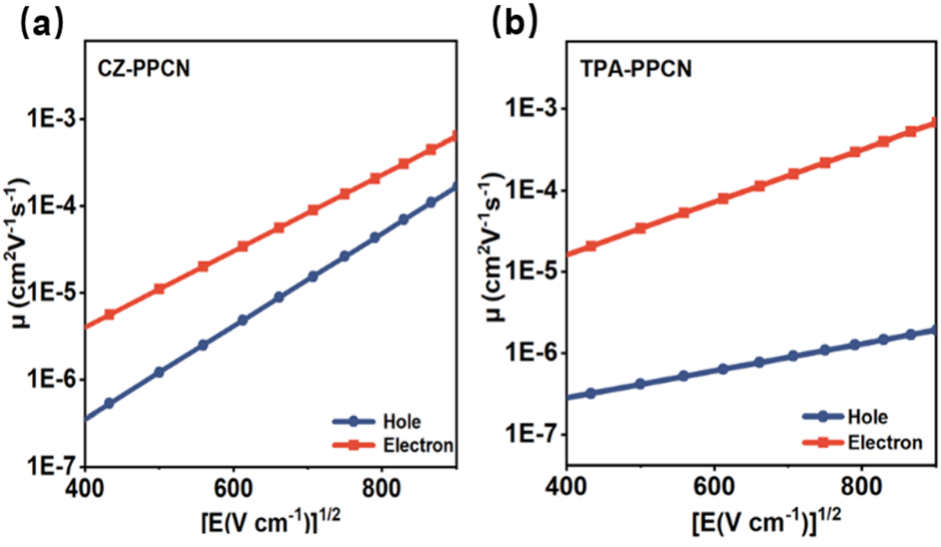
Charge mobilities *versus* electric field curves for (a) CZ-PPCN and (b) TPA-PPCN. Configuration of the hole-only device: ITO/HATCN (20 nm)/EML (80 nm)/HATCN (20 nm)/Al (100 nm). Configuration of the electron-only device: ITO/LiF (1 nm)/TPBi (10 nm)/EML (80 nm)/LiF (1 nm)/Al (100 nm).

Balanced carrier mobility is critical for OLEDs as it ensures uniform recombination of electrons and holes within the emissive layer, thereby enhancing both luminous efficiency and device stability. To further investigate the charge transport properties of the two emitters, hole-only and electron-only devices were fabricated. As shown in Fig. 4, under an electric field of 5.0 × 10^5^ V cm^−1^, CZ-PPCN exhibited hole and electron mobilities of 1.55 × 10^−5^ cm^2^ V^−1^ s^−1^ and 5.67 × 10^−5^ cm^2^ V^−1^ s^−1^, respectively, while TPA-PPCN demonstrated values of 7.65 × 10^−7^ cm^2^ V^−1^ s^−1^ and 1.13 × 10^−4^ cm^2^ V^−1^ s^−1^. Particularly, both materials displayed electron mobilities surpassing their hole mobilities—a deviation from conventional OLED materials where hole mobility typically dominates,^41^ with this trend being particularly pronounced in TPA-PPCN. As anticipated, CZ-PPCN exhibited significantly more balanced carrier transport than TPA-PPCN, which underpins its better electroluminescent performance. This discrepancy is closely tied to their distinct molecular packing and intermolecular interactions, as revealed by single-crystal analysis.

### Device performance

**Fig. 5:**
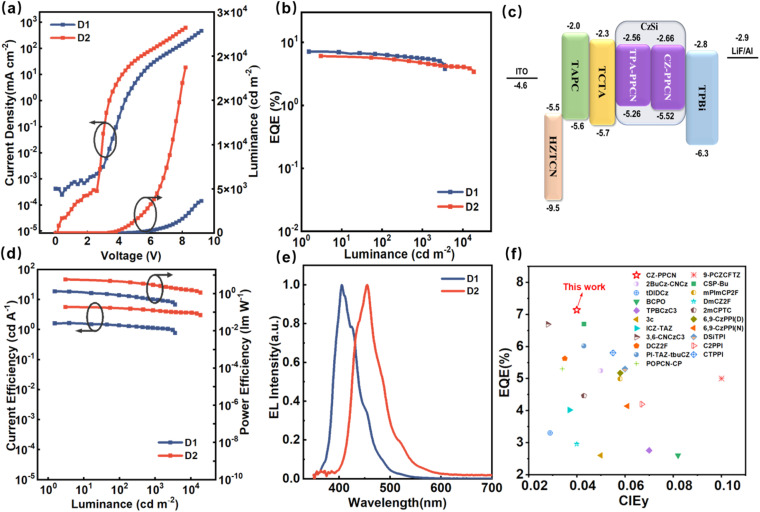
(a) Current density–voltage–luminance curves, (b) EQE–luminance curves, (c) device structures and energy levels, (d) current efficiency–luminance–power efficiency curves, and (e) EL spectra of doped OLEDs with 25 wt% emitters (CZ-PPCN and TPA-PPCN for D1 and D2). (f) Performance comparison of NUV electroluminescent devices (EL peak ≤ 410 nm) based on the HLCT mechanism (“N” for a non-doped device and “D” for a doped device).

To systematically investigate the EL performance of the molecules, we fabricated two non-doped devices (denoted as N1 with CZ-PPCN and N2 with TPA-PPCN as the emission layers, EMLs) using the optimized architecture ITO/HATCN (20 nm)/TAPC (35 nm)/TCTA (5 nm)/mCP (5 nm)/EML (20 nm)/TPBi (35 nm)/LiF (1 nm)/Al (100 nm). In this structure: HATCN and LiF serve as the hole- and electron-injection layers, respectively. TAPC (4,4′4′′-tri(9-carbazoyl)triphenylamine) functions as the hole-transporting layer (HTL). TCTA (4,4′,4′′-tris(carbazol-9-yl)triphenylamine) acts as the electron-blocking layer (EBL). mCP (1,3-di(carbazol-9-yl)benzene), with its high triplet energy (T_1_), serves as a buffer layer between the HTL and EML. TPBi (1,3,5-tris(*N*-phenylbenzimidazol-2-yl)benzene) is employed as the electron-transporting layer. Device performance data are summarized in Table 1 and Fig. S14.† Compared to their PL spectra in neat films, the EL spectra of both devices exhibit a blue-shift, attributed to restricted molecular motion in the solid state. The low turn-on voltages (2.8 V for N1 and N2) confirm rational energy-level alignment in the device architecture. While the non-doped devices achieved EQEs of 6.42% (N1) and 4.16% (N2), their color purity remained suboptimal. To enhance EL performance, doped devices were fabricated by incorporating CZ-PPCN and TPA-PPCN as dopants (25 wt%) into the CzSi host matrix, which exhibits balanced hole/electron transport capabilities to ensure uniform carrier distribution within the emissive layer. The optimized device structure is as follows (denoted as D1 with CZ-PPCN and D2 with TPA-PPCN as the emission layers, EMLs): ITO/HATCN (20 nm)/TAPC (35 nm)/TCTA (5 nm)/EMLs (20 nm)/TPBi (35 nm)/LiF (1 nm)/Al (100 nm). Detailed data are reported in Fig. 5. Doping significantly improved device performance, with CZ-PPCN achieving an EL emission maximum *λ*_EL_ at 405 nm (FWHM = 51 nm) and an EQE of 7.14% (CIE: 0.159, 0.040), while TPA-PPCN exhibited a red-shifted *λ*_EL_ at 455 nm (FWHM = 61 nm, CIE: 0.176, 0.105) and an EQE of 6.07%, attributed to stronger intramolecular charge-transfer (ICT) effects.

**Table 1:** Electroluminescent properties of OLEDs

Devices	Emissive layer	*V*_on_ a [V]	CE_max_^b^ [cd A^−1^]	PE_max_^c^ [lm W^−1^]	*L*_max_ d [d m^−2^]	EQE_max_^e^ [%]	*λ*_EL_ f [nm]	CIE (*x*, *y*)
N1	CZ-PPCN	2.8	4.27	4.00	12 835	6.42	430	(0.154, 0.075)
N2	TPA-PPCN	2.8	8.84	8.71	27 090	4.16	485	(0.168, 0.330)
D1	CzSi: CZ-PPCN	3.6	1.64	1.33	3630	7.14	405	(0.159, 0.040)
D2	CzSi: TPA-PPCN	2.8	5.62	5.88	18 731	6.07	455	(0.149, 0.105)

a *V*_on_: turn-on voltage at 1 cd m^−2^.

b CE_max_: maximum current efficiency.

c PE_max_: maximum power efficiency.

d *L*_max_: maximum luminance.

e EQE_max_: maximum value of external quantum efficiency.

f EL: electroluminescence peak wavelength.

Specifically, the calculated exciton utilization efficiencies (EUEs) for N1 and N2 reached 37.7% and 26%, respectively. The EUE of 37.7% for N1 stands as the primary evidence demonstrating the capability of the proposed mechanism to overcome the spin-statistical limitation for high-efficiency NUV-OLEDs. The performance of N2, achieving an EUE marginally above the 25% threshold, provides further supportive evidence for the role of RISC. As shown in Fig. 5f, through systematic comparison of NUV emitters based on the HLCT mechanism (400 nm ≤ *λ*_EL_ ≤ 410 nm), the CZ-PPCN molecule demonstrates unique advantages in achieving high color purity while maintaining high EQE (Table S5†).

## Conclusions

In summary, we successfully synthesized and characterized two simple D–π–A type emitters, CZ-PPCN and TPA-PPCN, featuring benzonitrile as the acceptor and carbazole/triphenylamine as donors. Theoretical calculations, Lippert–Mataga solvent modeling, large Δ*E*_S1T1_ values derived from low-temperature spectroscopy, and nanosecond-scale lifetimes in neat films collectively confirm the HLCT nature of the S_1_ excited state. Single-crystal analysis further elucidated the intermolecular interactions, with the cyano group playing a critical role in establishing robust charge transport pathways. Both materials exhibit electron mobilities exceeding hole mobilities, attributed to continuous transport channels enabled by moderate π–π stacking and abundant intermolecular interactions. Specifically, CZ-PPCN demonstrated superior balanced carrier mobility (*μ*_h_ = 1.55 × 10^−5^ cm^2^ V^−1^ s^−1^; *μ*_e_ = 5.57 × 10^−5^ cm^2^ V^−1^ s^−1^), enabling the non-doped device to achieve better performance with EQEs of 6.42%. This doped device demonstrates unique synergy, simultaneously achieving high color purity (CIE_*y*_ = 0.04) and a high EQE of 7.14%. This combined performance advantage makes it exceptional among comparable devices based on the HLCT mechanism (400 nm ≤ *λ*_EL_ ≤ 410 nm).

## Author contributions

L. Zhang completed all the experiments and wrote the first draft of the paper with the help of S. Xue. C. Ma contributed to the preparation and characterization of the OLED devices. X. Wang contributed to editing the article. Y. Zhou, J. Song, M. Sun, and Q. Sun helped to review and revise it. S. Zhang participated in the quantum chemical calculations. S. Xue and W. Yang guided the project and data analysis, and edited and revised the final manuscript.

## Conflicts of interest

There are no conflicts to declare.

## Supplementary Material

SC-OLF-D5SC03458B-s001

SC-OLF-D5SC03458B-s002

## Data Availability

The data supporting this article have been included as part of the ESI.† Additional data are available from the corresponding author upon reasonable request. Crystallographic data can be obtained free of charge from The Cambridge Crystallographic Data Centre *via* Structural Chemistry Data, Software, and Insights | CCDC. Supplementary crystallographic data for CCDC File No. 2446929 and 2447271 (CIF).
