# Supplementary material for: Efficient near-ultraviolet (NUV) electroluminescence based on a benzonitrile acceptor HLCT material with balanced carrier mobilities and high color purity
Source: Chem Sci. 2025 Jul 17;16(32):14478–84. doi: 10.1039/d5sc03458b (PMC12268602; doi:10.1039/d5sc03458b)
Supplement: SC-016-D5SC03458B-s001 [file SC-016-D5SC03458B-s001.pdf]

Supplementary Information (SI) for Chemical Science.
This journal is © The Royal Society of Chemistry 2025

Supporting Information

Efficient near-ultraviolet (NUV) electroluminescence based on benzonitrile acceptor HLCT material with balanced carrier mobilities and high color purity

Li Zhang[a], Chenglin Ma[a], Xin Wang[a], Yannan Zhou[a], Jingru Song[a], Mizhen Sun[a], Qikun Sun[a], Shi-Tong Zhang[b], Wenjun Yang[a], Shanfeng Xue[a]*

[a]*State Key Laboratory of Advanced Optical Polymer and Manufacturing Technology, Qingdao University of Science and Technology, 53-Zhengzhou Road, Qingdao 266042, P. R. China.*

[b]*State Key Laboratory of Supramolecular Structure and Materials, Department of Chemical Engineering and Applied Chemistry, College of Chemistry, Jilin University, Changchun 130012, P. R. China.*

**Corresponding author. E-mail:* sfxue@qust.edu.cn

CONTENTS

S1 General Measurements

S2 Synthesis and routines

S3 Supplementary figures and tables

S1 General Measurements

1. General Measurements

The ^{1}H NMR and ^{13}C NMR spectrum was recorded on a Bruker AC500 spectrometer at 500 and 126 MHz, respectively, using deuterated chloroform ($CDCl_3$) as solvents. The chemical shift for each signal was reported in ppm units with tetramethylsilane (TMS) as a standard internal reference. The MALDI-TOF-MS mass spectra were recorded using an AXIMA-CFRTM instrument. UV-vis absorption and fluorescence spectra of solution and film were recorded by a Hitachi U-4100 spectrophotometer and a Hitachi F-4600 spectrophotometer, respectively. An FLS980 spectrometer measured photoluminescence quantum yield. The lifetime was measured on an Edinburgh FLS-1000 spectrometer with an EPL-310 optical laser. In the solution-related tests covered in this work, the solution concentration is 10^{-5} M.

2. Electrochemical Measurements

Cyclic voltammetry was performed with a BAS 100 W Bioanalytical system, using a glass carbon disk (Φ = 3 mm) as the working electrode, a platinum wire as the auxiliary electrode with a porous ceramic wick, and Ag/Ag^{+} as the reference electrode, standardized for the redox couple ferricinium/ferrocene. All solutions were purged with a nitrogen stream for 10 min before measurement. The procedure was performed at room temperature, and a nitrogen atmosphere was maintained over the solution during the measurements. The energy levels of HOMO and LUMO are calculated according to the Formula below:

$$\mathrm{HOMO} = -(E_{ox}\ \mathrm{vs.}\ Ag/Ag^{+} - E_{1/2}{}^{+}\ \mathrm{vs.} Ag/Ag^{+} + 4.8)\ \mathrm{eV}$$

$$\mathrm{LUMO} = -(E_{red}\ \mathrm{vs.}\ Ag/Ag^{+} - E_{1/2}{}^{-}\ \mathrm{vs.} Ag/Ag^{+} + 4.8)\ \mathrm{eV}$$

The E_{ox} vs. Ag/Ag^{+} and E_{red} vs. Ag/Ag^{+} are oxidation and reduction onset potentials relative to the Ag/Ag^{+} electrode. Ferrocene was used as an internal standard. $E_{1/2}{}^{+}$ vs. Ag/Ag^{+} and $E_{1/2}{}^{-}$ vs. Ag/Ag^{+} are half-wave potentials of Fc^{+} /Fc^{-} obtained from positive and negative CV scans, respectively.

3. Thermal Stability Measurements

The differential scanning calorimetry (DSC) dates are from the NETZSCH (DSC-204) instrument, which was heated to 20-300 °C at a heating rate of 10 °C /min and a nitrogen flow rate of 80 mL/min. The material was thermal gravimetrically analyzed (TGA) using a Perkin-Elmer thermal analysis system from 40 to 800 °C at a heating rate of 10 °C/min under the nitrogen atmosphere.

4. Device fabrication and characterization

Before the measurement, vacuum sublimation obtained the target molecules as neat products. TPBi used in the device was purchased from Jilin OLED Material Tech Co., Ltd., and HATCN, TCTA, TAPC and mCP were purchased from Xi'an Polymer Light Technology Corp. ITO-coated glass with a sheet resistance of 10 Ω square^{-1} and was used as the substrate. The pre-treatment of ITO glass included a routine chemical cleaning using detergent and alcohol in sequence, dried in an oven at 120 °C. After the oxygen plasma was cleaned for 7 min and finally transferred to a vacuum deposition system with a base pressure greater than 1.6×10^{-4} Pa for organic and metal deposition. The current-voltage–luminance characteristics were measured using a Keithley source measurement unit (Keithley 2450 and LS-160). The electroluminescent (EL) spectra and Commission Internationale de l'Eclairage (CIE) coordinates of these devices were measured with a Flame-S (Serial Number: FLMS16791). EQEs were calculated from the luminance, current density, and EL spectrum.

5. Efficiency Measurement:

The EUE was calculated by the following Formula S1:

$$\eta_{EQE} = \eta_{rec} \cdot \Phi_{PL} \cdot \eta_{EUE} \cdot \eta_{out} \quad \text{(Formula S1)}$$

Where η_{rec} is the ratio of electron hole recombination (ideally, if electrons and holes completely recombine to form excitons in the EML layer, this value is 100%), Φ_{PL} is the PLQY of non-doped neat films, and η_{EUE} is the exciton utilization efficiency η_{out} is the light out-coupling efficiency, which is 20% for glass substrate and randomly

stacked film.

S2 Synthesis and Routines

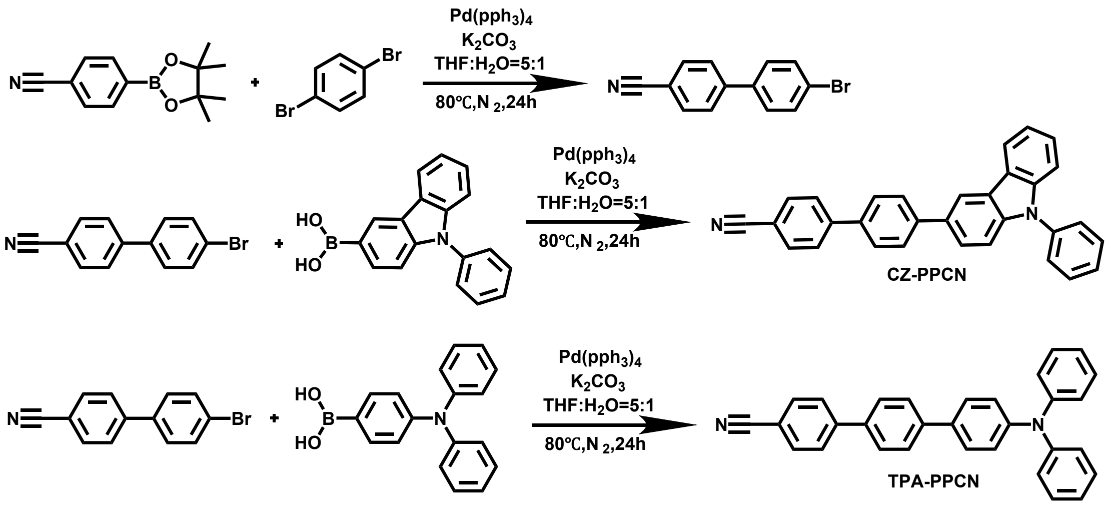

Fig. S1 Target compound synthesis routes.

(1) Synthesis of 4'-bromo-[1,1'-biphenyl]-4-carbonitrile

4-(4,4,5,5-tetramethyl-1,3,2-dioxaborolan-2-yl) benzonitrile (2.66 g, 11.60 mmol), 1,4-dibromo-2,5-difluorobenzene (3.29 g, 13.90 mmol), potassium carbonate (2.40 g, 17.40 mmol), tetrahydrofuran (50 mL) and deionized water (10 mL), with Pd (PPh3)$_4$ (0.53 g, 0.46 mmol) acting as catalyst was refluxed at 90 °C for 24 h under nitrogen. After the mixture was cooled down, 40 mL deionized water was added to the resulting solution and the mixture was extracted with dichloromethane for several times. The organic phase was dried over anhydrous magnesium sulphate. After filtration and solvent evaporation, the given residue was purified through silica gel column chromatography using ethyl petroleum ether/dichloromethane as eluent to give the product as white solid (2.37 g, 79.0 %).

(2) Synthesis of 4'-(9-phenyl-9H-carbazol-3-yl)-[1,1'-biphenyl]-4-carbonitrile (CZ-PPCN)

(9-phenyl-9H-carbazol-3-yl) boronic acid (1.83 g, 7.10 mmol), 4'-bromo-2',5'-difluoro-[1,1'-biphenyl]-4-carbonitrile (2.46 g, 8.52 mmol), potassium carbonate (1.47 g, 10.65 mmol), tetrahydrofuran (50 mL) and deionized water (10 mL), with Pd (PPh3)4 (0.32 g, 0.28 mmol) acting as catalyst was refluxed at 90 °C for 24 h under nitrogen. After the mixture was cooled down, 40 mL deionized water was added to the resulting solution and the mixture was extracted with dichloromethane for several times. The

organic phase was dried over anhydrous magnesium sulphate. After filtration and solvent evaporation, the given residue was purified through silica gel column chromatography using ethyl petroleum ether/dichloromethane as eluent to give the product as white solid (2.4 g, 80.0 %). ^{1}H NMR (500 MHz, Chloroform-*d*) δ 8.41 – 8.39 (m, 1H), 8.21 (dt, J = 7.8, 1.0 Hz, 1H), 7.86 – 7.82 (m, 2H), 7.75 (d, J = 1.2 Hz, 4H), 7.73 – 7.68 (m, 3H), 7.66 – 7.58 (m, 4H), 7.52 – 7.48 (m, 2H), 7.45 – 7.43 (m, 2H), 7.32 (ddd, J = 8.0, 5.0, 3.1 Hz, 1H). ^{13}C NMR (126 MHz, CDCl3) δ 145.32, 142.39, 141.44, 140.65, 137.56, 137.17, 132.67, 132.32, 130.00, 127.91, 127.67, 127.64, 127.52, 127.09, 126.34, 125.32, 124.02, 123.40, 120.41, 120.24, 119.06, 118.80, 110.76, 110.22, 110.06. MALDI-TOF MS (mass m/z): calcd for $C_{30}H_{20}N_2$, 420.1626; found, 420.1627 $[M + H]^+$.

(3) Synthesis of 4''-(diphenylamino)-[1,1':4',1''-terphenyl]-4-carbonitrile (TPA-PPCN)

(4-(diphenylamino) phenyl) boronic acid (1.83 g, 7.10 mmol), 4'-bromo-2',5'-difluoro-[1,1'-biphenyl]-4-carbonitrile (2.46 g, 8.52 mmol), potassium carbonate (1.47 g, 10.65 mmol), tetrahydrofuran (50 mL) and deionized water (10 mL), with Pd $(PPh_3)_4$ (0.32 g, 0.28 mmol) acting as catalyst was refluxed at 90 °C for 24 h under nitrogen. After the mixture was cooled down, 40 mL deionized water was added to the resulting solution and the mixture was extracted with dichloromethane for several times. The organic phase was dried over anhydrous magnesium sulphate. After filtration and solvent evaporation, the given residue was purified through silica gel column chromatography using ethyl petroleum ether/dichloromethane as eluent to give the product as white solid (2.37 g, 79.0 %). ^{1}H NMR (500 MHz, Chloroform-*d*) δ 7.73 (s, 4H), 7.70 – 7.64 (m, 4H), 7.53 – 7.50 (m, 2H), 7.31 – 7.26 (m, 4H), 7.17 – 7.13 (m, 6H), 7.05 (tt, J = 7.4, 1.2 Hz, 2H). ^{13}C NMR (126 MHz, CDCl3) δ 147.70, 147.57, 145.21, 141.04, 137.36, 133.74, 132.66, 129.37, 127.70, 127.60, 127.50, 127.24, 124.64, 123.64, 123.20, 119.97, 119.02, 110.80，53.46. MALDI-TOF MS (mass m/z): calcd for $C_{30}H_{22}N_2$, 423.1858; found, 422.1777 $[M + H]^+$.

S3 Supplementary figures and tables

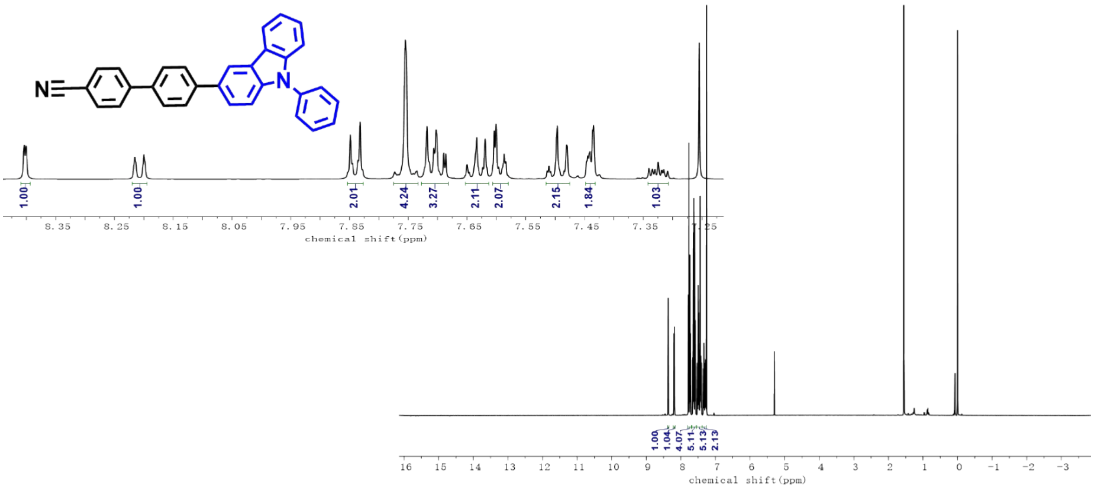

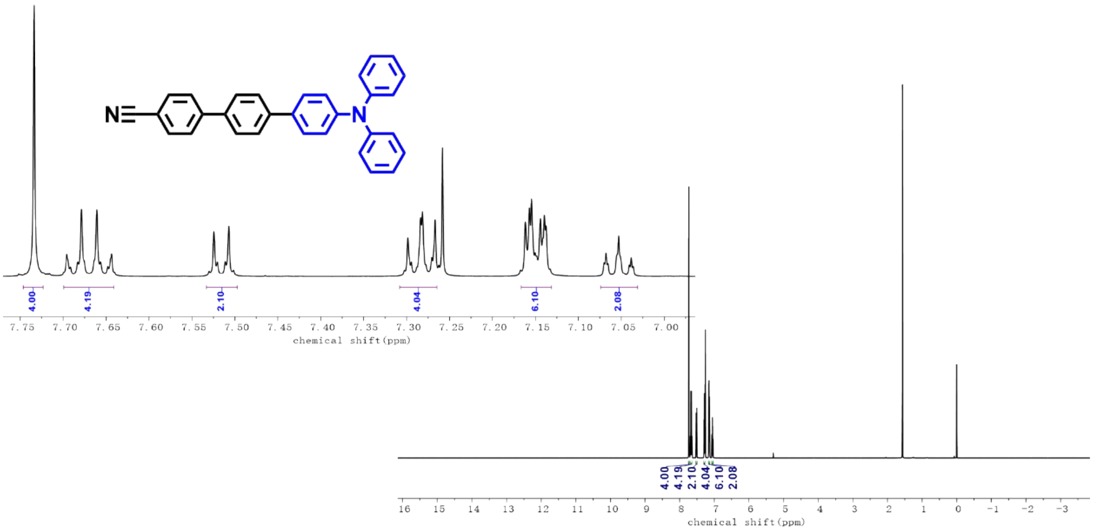

Fig. S2 ^{1}H-NMR Spectrum of CZ-PPCN and TPA-PPCN in $CDCl_3$.

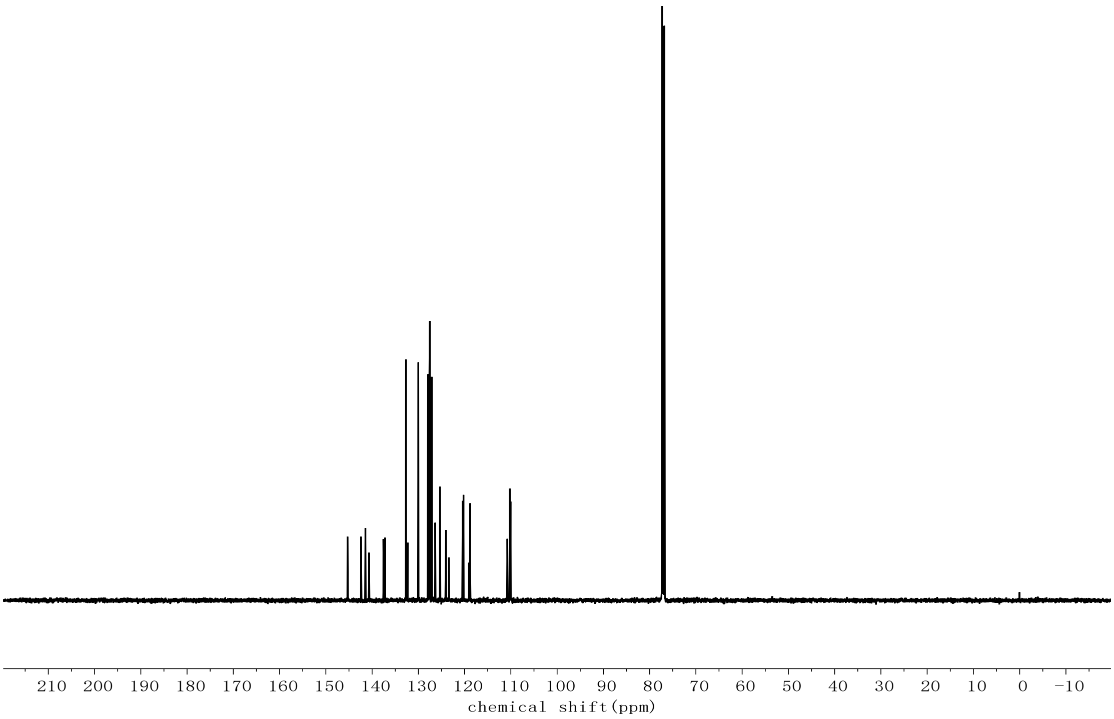

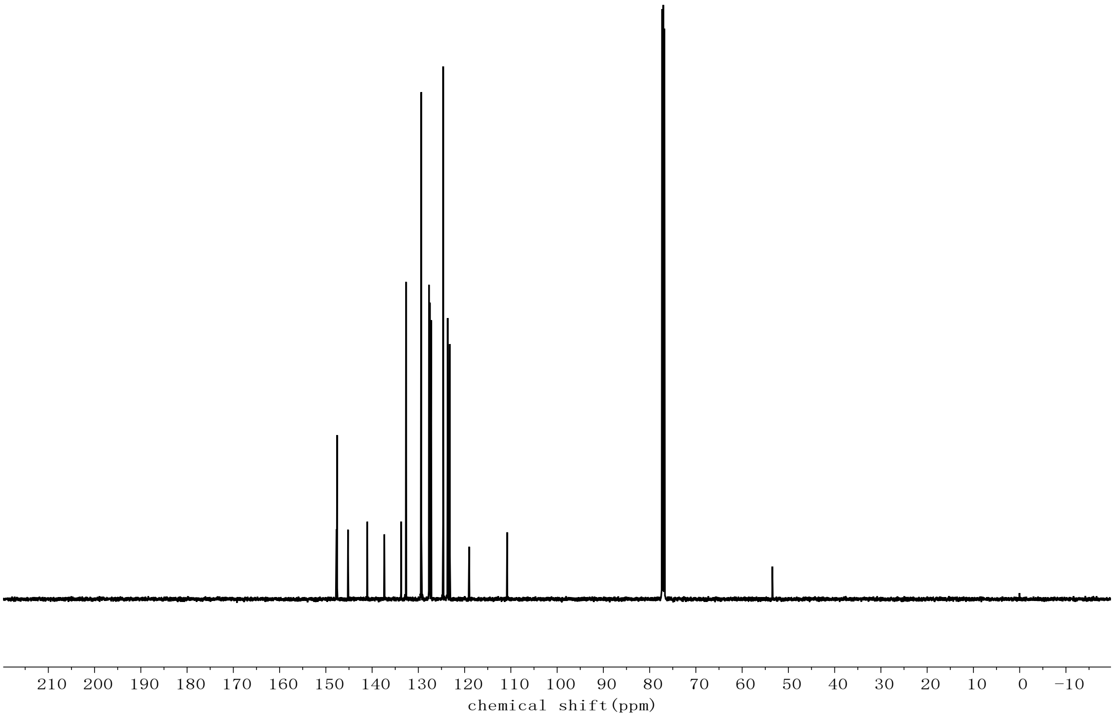

Fig. S3 ^{13}C-NMR Spectrum of CZ-PPCN and TPA-PPCN in $CDCl_3$.

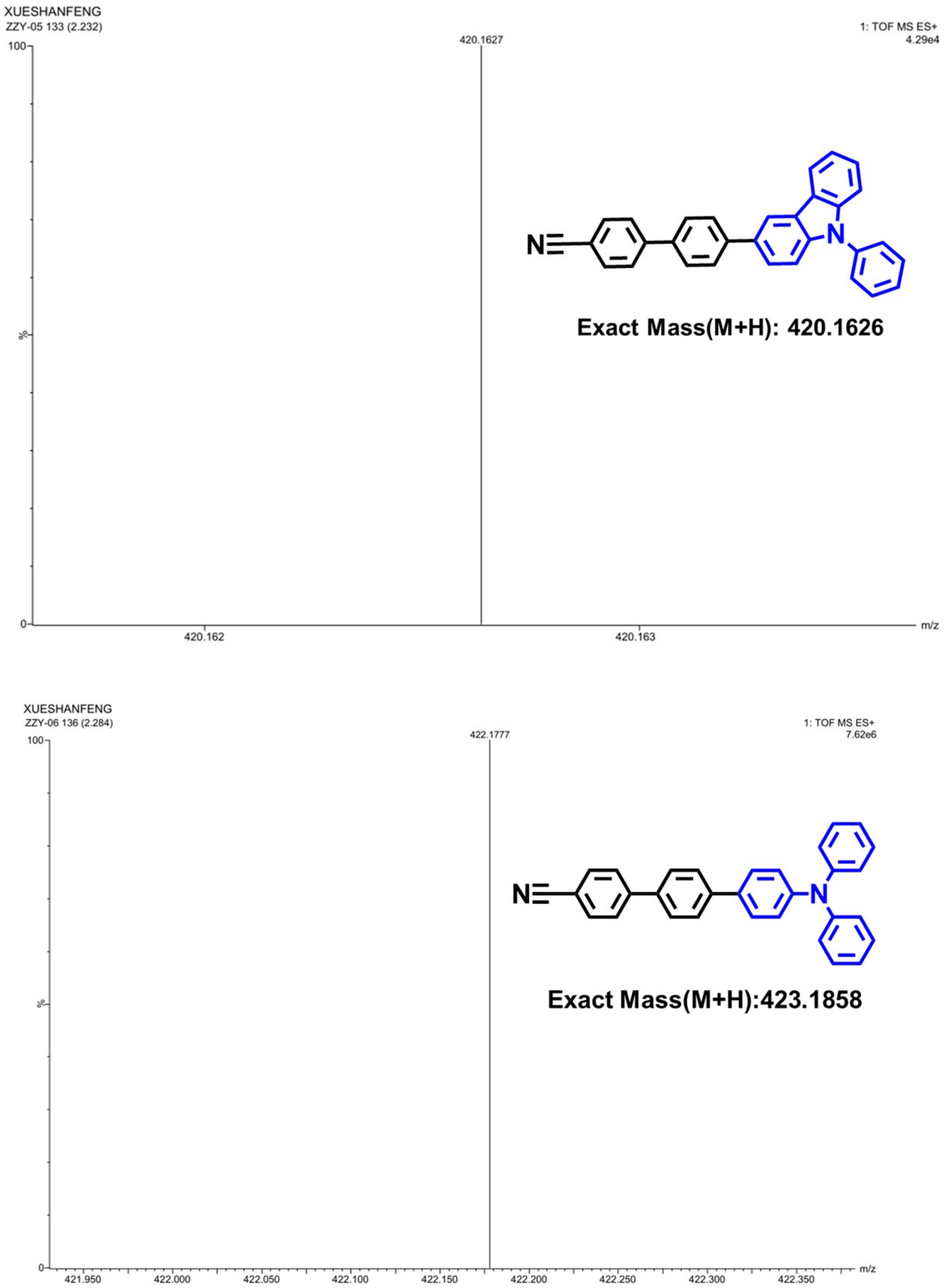

Fig. S4 Mass Spectrum $(M+H)^+$ of CZ-PPCN and TPA-PPCN.

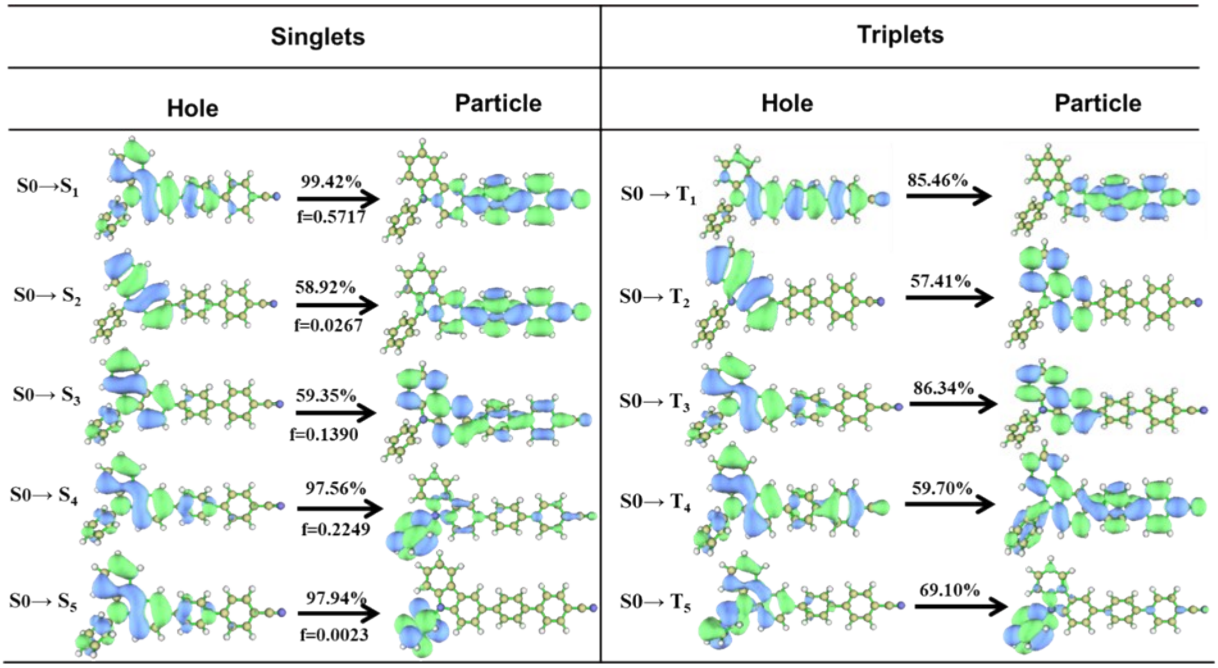

Fig. S5 The natural transition orbitals (NTOs) of the singlet (S_{1-5}) and triplet (T_{1-5}) excited states for CZ-PPCN.

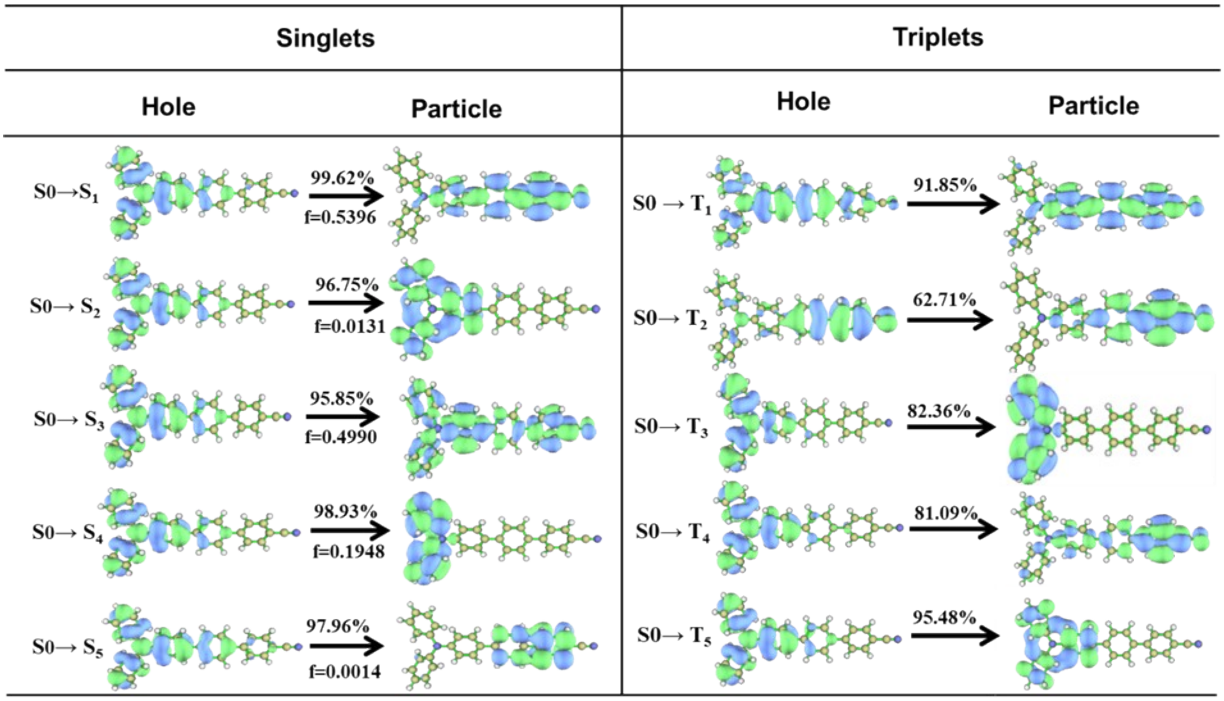

Fig. S6 The natural transition orbitals (NTOs) of the singlet (S_{1-5}) and triplet (T_{1-5}) excited states for TPA-PPCN.

Table S1 The singlet and triplet energy levels for CZ-PPCN and TPA-PPCN.

CZ-PPCN				TPA-PPCN			
S Excited States	[eV]	T Excited States	[eV]	S Excited States	[eV]	S Excited States	[eV]
S1	3.402	T1	2.756	S1	3.026	T1	2.561
S2	3.81	T2	3.161	S2	3.85	T2	3.068
S3	3.967	T3	3.255	S3	3.902	T3	3.195
S4	4.174	T4	3.426	S4	4.064	T4	3.392
S5	4.285	T5	3.567	S5	4.117	T5	3.47
S6	4.355	T6	3.675	S6	4.213	T6	3.61
S7	4.397	T7	3.852	S7	4.262	T7	3.87
S8	4.42	T8	3.958	S8	4.416	T8	3.884
S9	4.524	T9	4.047	S9	4.442	T9	3.978
S10	4.651	T10	4.078	S10	4.705	T10	3.999

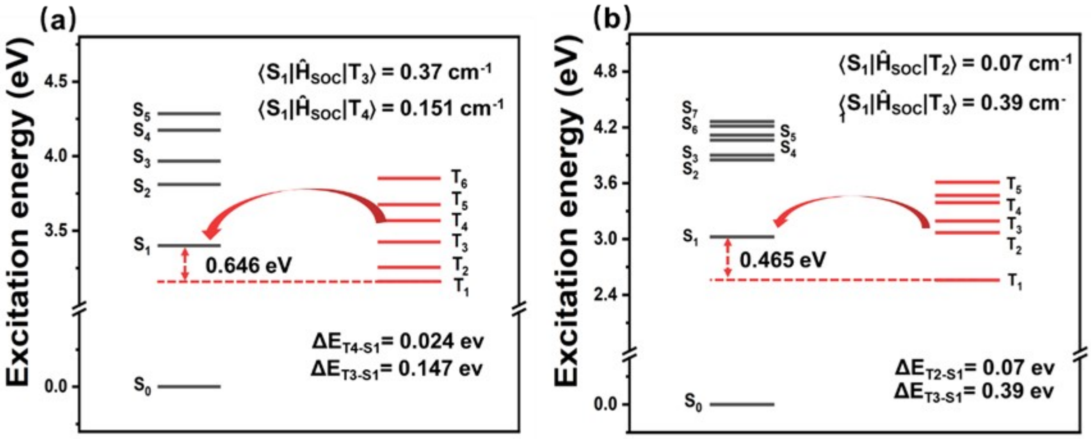

Fig. S7 The plots of energy levels of singlet and triplet states of CZ-PPCN and TPA-PPCN.

Table S2. LE/CT ratio of CZ-PPCN and TPA-PPCN.

Compound	LE [%]	CT [%]
CZ-PPCN	23.95	76.05
TPA-PPCN	18.02	81.08

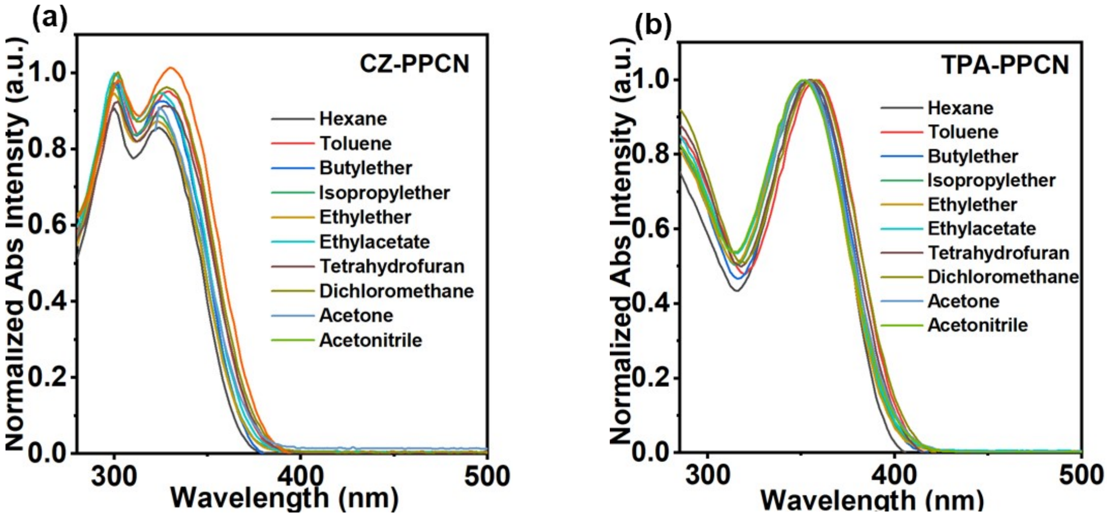

Fig. S8 The UV–vis absorption spectra of CZ-PPCN and TPA-PPCN in different solvents.

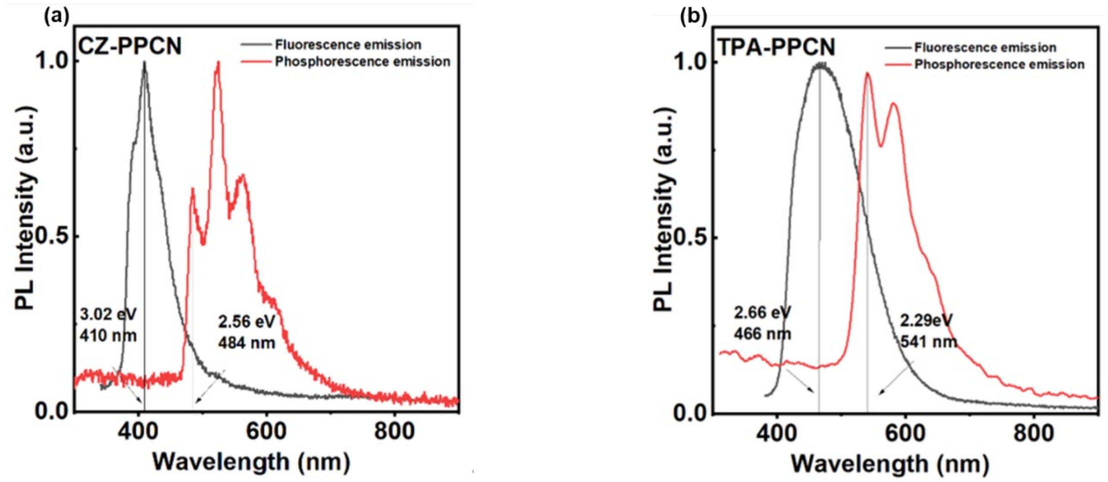

Fig. S9 The fluorescence and phosphorescence spectra in dilute THF solution at 77 K.

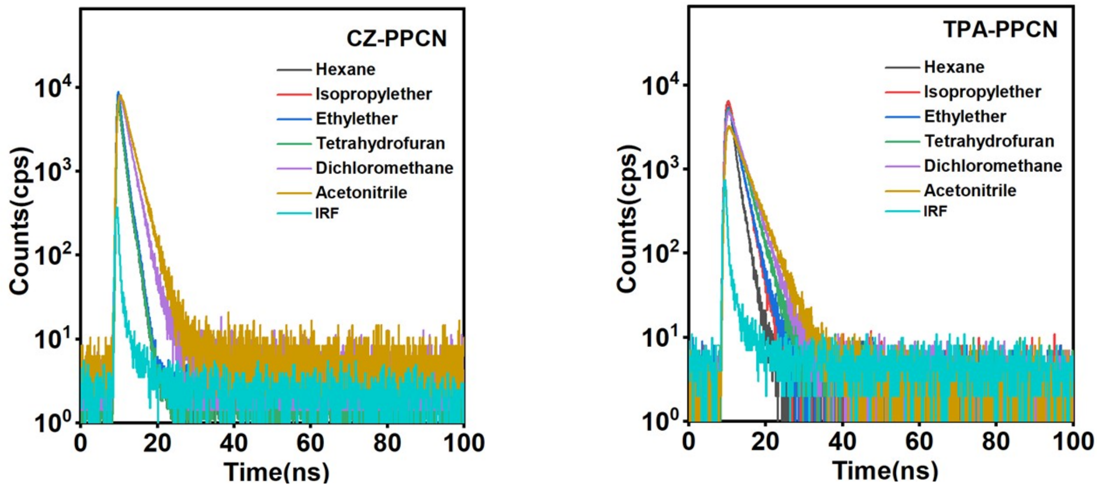

Fig. S10 The transient PL decay spectra of CZ-PPCN and TPA-PPCN in different solvents.

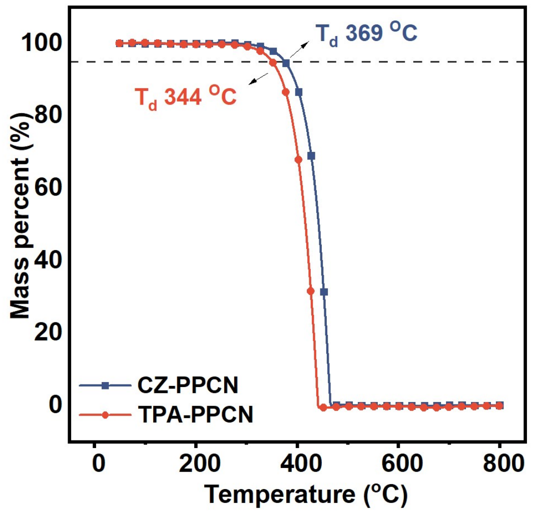

Fig. S11 The thermogravimetric analysis (TGA) curves.

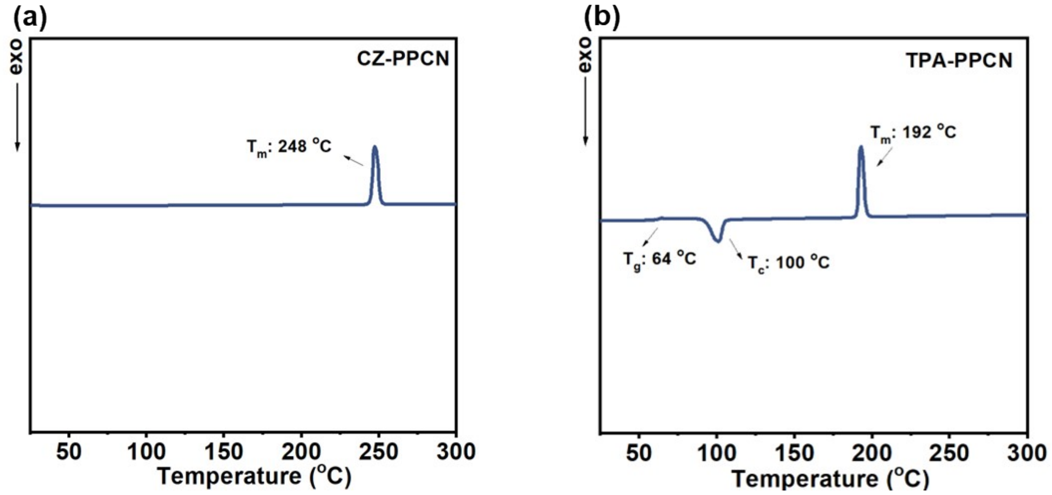

Fig. S12 The differential scanning calorimeter (DSC).

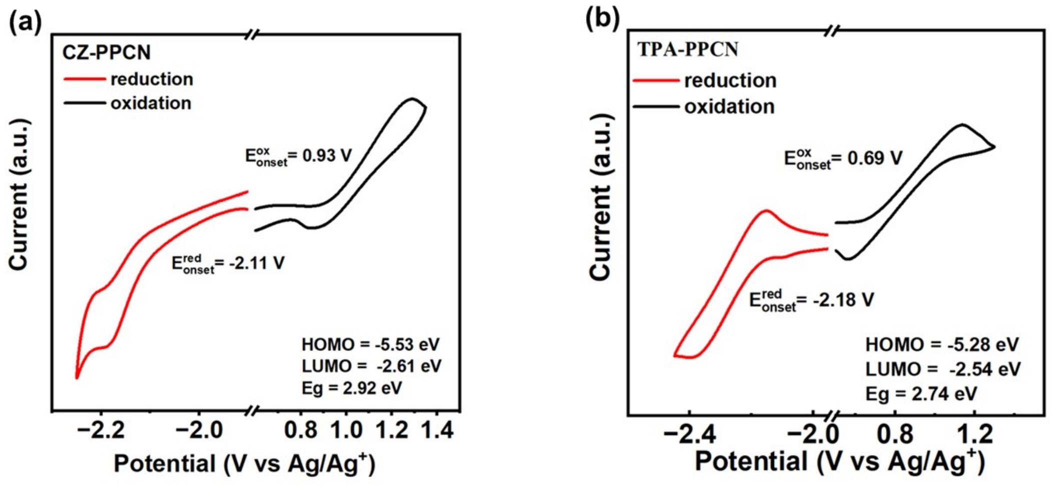

Fig. S13 The cyclic voltammetry (CV) curves of CZ-PPCN and TPA-PPCN.

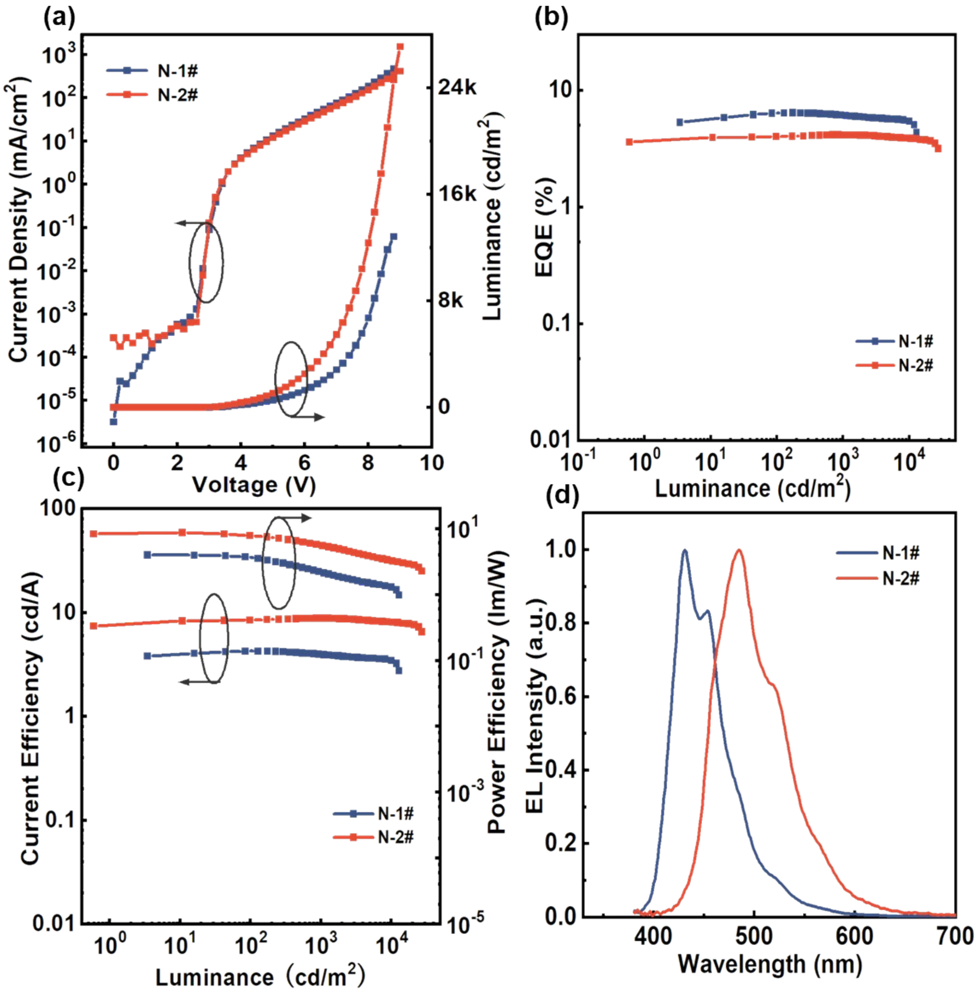

Fig. S14 Non-doped OLED performances of CZ-PPCN and TPA-PPCN. (a) Current density-voltage-luminance curves, (b) EQE-luminance curves, (c) current efficiency–luminance-power efficiency curves, and (d) EL spectra.

Table S3 Key thermal and photophysical properties of emitters.

compounds	λ_{abs} $^{(a)}$[nm]	λ_{PL} $^{(b)}$[nm]	$\phi_{PL}$$^{(c)}$[%]		$\tau^{(d)}$[ns]		T_d[℃]
	THF	THF/ film 1/film 2	THF	Neat Film	THF	Neat film	
CZ-PPCN	300/330	430/443/426	91.5	85	1.725	3.41	369
TPA-PPCN	282/354	472/489/461	97.8	80	2.515	5.54	344

(a) λ_{abs}: absorption maximum in the 10^{-5} M THF; (b) λ_{PL}: emission peak in the 10^{-5} M THF; the neat film and the doped film; (c) PLQY is the quantum efficiency of photoluminescence in the 10^{-5} M THF and neat films; (d) fluorescence lifetime.

Table S4 Crystal data for CZ-PPCN and TPA-PPCN.

Compound	CZ-PPCN	TPA-PPCN
Chemical formula	$C_{31}H_{20}N_2$	$C_{31}H_{22}N_2$
Formula weight	420.49	422.51
Crystal system	monoclinic	triclinic
a/Å	18.6571(8)	8.147
b/Å	16.1840(7)	9.625
c/Å	7.4554(3)	16.714
α/°	90	97.07
β/°	91.619(2)	100.55
γ/°	90	113.8
Unit cell volume/ Å3	2250.23(16)	1150.2
Temperature/K	273	273
Space group	P 21/c	P -1
Z	4	2
Density (calculated) /g cm^{-3}	1.241	1.22
F(000)	880.0	444.0
Index ranges	-21 =< h =< 22<br>-19 =< k =< 19<br>-8 =< l =< 8	-21 =< h =< 22<br>-19 =< k =< 19<br>-8 =< l =< 8
Reflections measured	3971	4094
Completeness to theta = 72.13°	99.2%	98.9%
Min. and max. transmission	0.935 and 0.956	0.936 and 0.957
Data / restraints / parameters	3941/0/298	4050/0/298
Goodness-of-fit on F^2	1.061	1.061
Final R_1 values (all data)	0.0541	0.0799
Final $wR(F^2)$ values (all data)	0.1457	0.2713
CCDC number	2446929	2447271

Table S5 Device Performances Comparison for NUV OLEDs with EL Peak ≤ 410 nm.

Emmitter	EL peak (nm)	EQE_{max} (%)	CIE-y	Reference/year
**2BuCz-CNCz**	408	5.24	0.050	[1]/2021
**tDIDCz**	402	3.30	0.029	[2]/2020
**BCPO**	408	2.6	0.082	[3]/2020
**TPBCzC3**	405	2.76	0.07	[4]/2020
**3c**	408	2.6	0.05	[5]/2020
**ICZ-TAZ**	406	4.02	0.037	[6]/2022
**3,6-CNCzC3**	407	6.69	0.028	[7]/2024
**DCZ2F**	404	5.62	0.035	[8]/2023
**PI-TAZ-tbuCZ**	407	6.01	0.043	[9]/2022
**POPCN-CP**	404	5.3	0.034	[10]/2022
**9-PCZCFTZ**	404	5.0	0.1	[11]/2024
**CSP-Bu**	404	6.7	0.043	[12]/2023
**mPImCP2F**	404	4.99	0.058	[13]/2025
**DmCZ2F**	403	2.96	0.040	[13]/2025
**2Mcptc**	406	4.46	0.043	[14]/2025
**6,9-CzPPI (D)**	406	5.17	0.058	[15]/2023
**6,9-CzPPI (N)**	408	4.14	0.061	[15]/2023
**DSiTPI**	406	5.3	0.06	[16]/2021
**C2PPI**	406	4.2	0.067	[17]/2022
**CTPPI**	404	5.8	0.55	[18]/2022

Reference

1 H. Zhang, G. Li, X. Guo, K. Zhang, B. Zhang, X. Guo, Y. Li, J. Fan, Z. Wang, D. Ma and B. Z. Tang, *Angew Chem Int Ed*, 2021, **60**, 22241–22247.

2 H. L. Lee, W. J. Chung and J. Y. Lee, *Small*, 2020, **16**, 1907569.

3 S. Yan, M. Qin, C. Shen, L. Niu and Y. Zhang, *Synth. Met.*, 2020, **263**, 116368.

4 P. Han, C. Lin, D. Ma, A. Qin and B. Z. Tang, *ACS Appl. Mater. Interfaces*, 2020, **12**, 46366–46372.

5 V. Joseph, K. R. J. Thomas, S. Sahoo, M. Singh and J.-H. Jou, *Opt. Mater.*, 2020, **108**, 110159.

6 X. He, J. Lou, B. Li, H. Wang, X. Peng, G. Li, L. Liu, Y. Huang, N. Zheng, L. Xing, Y. Huo, D. Yang, D. Ma, Z. Zhao, Z. Wang and B. Z. Tang, *Angew Chem Int Ed*, 2022, **61**, e202209425.

7 H. Qi, S. Wang, Z. Gao, D. Xie, J. Li, Y. Liu, S. Xue, S. Ying, D. Ma and S. Yan, *ACS Mater Lett.*, 2024, **6**, 3844–3853.

8 Y. Huo, J. Lv, M. Wang, Z. Duan, H. Qi, S. Wang, Y. Liu, L. Peng, S. Ying and S. Yan, *J. Mater. Chem. C*, 2023, **11**, 6347–6353.

9 L. Peng, Y. Huo, L. Hua, J. Lv, Y. Liu, S. Ying and S. Yan, *J. Mater. Chem. C*, 2022, **10**, 9621–9627.

10 J. Chen, H. Liu, J. Guo, J. Wang, N. Qiu, S. Xiao, J. Chi, D. Yang, D. Ma, Z. Zhao and B. Z. Tang, *Angew Chem Int Ed*, 2022, **61**, e202116810.

11 H. Zhou, R. Wang, M. Sun, Y. Zhou, L. Zhang, J. Song, Q. Sun, S.-T. Zhang, W. Yang and S. Xue, *Chem. Sci.*, 2024, **15**, 18601–18607.

12 S. Geng, Z. Liu, H. Li, Z. Zhong, X. J. Feng, Z. Zhao and H. Lu, *Adv. Opt. Mater.*, 2024, **12**, 2301344.

13 S. Wang, R. Zhang, R. Ding, H. Huang, H. Qi, Y. Liu, S. Ying, D. Ma and S. Yan, *Chem. Sci.*, 2025, **16**, 5518–5527.

14 P. Zou, Z. Yang, X. Dong, Z. Zhong, L. Xu, B. Z. Tang and Z. Zhao, *Chemistry A European J*, 2025, **31**, e202500644.

15 X. Wang, Z. Liu, S. Geng, Z. Zhong, H. Li, X. J. Feng, Z. Zhao and H. Lu, *J. Mater. Chem. C*, 2023, **11**, 5316–5323.

16 Y. Zheng, X. Zhu, Z. Ni, X. Wang, Z. Zhong, X. J. Feng, Z. Zhao and H. Lu, *Adv. Opt. Mater.*, 2021, **9**, 2100965.

17 Z. Zhong, Z. Liu, S. Geng, H. Li, X. J. Feng, Z. Zhao and H. Lu, *J. Mater. Chem. C*, 2023, **11**, 1733–1741.

18 Z. Zhong, X. Zhu, X. Wang, Y. Zheng, S. Geng, Z. Zhou, X. J. Feng, Z. Zhao and H. Lu, *Adv. Funct. Mater.*, 2022, **32**, 2112969.
